# Vitamin D Suppresses Ovarian Cancer Growth and Invasion by Targeting Long Non-Coding RNA CCAT2

**DOI:** 10.3390/ijms21072334

**Published:** 2020-03-27

**Authors:** Liye Wang, Shuang Zhou, Bin Guo

**Affiliations:** Department of Pharmacological and Pharmaceutical Sciences, College of Pharmacy, University of Houston, Houston, TX 77204, USA; lwang53@uh.edu (L.W.); goldenshuang1929@gmail.com (S.Z.)

**Keywords:** long non-coding RNA, CCAT2, vitamin D, cancer prevention, ovarian cancer

## Abstract

Ovarian cancer is the most deadly gynecologic cancer among women worldwide. Poor response to current treatment makes it necessary to discover new diagnostic biomarkers to detect the cancer early and develop new and effective prevention strategies. Calcitriol, the active metabolite of vitamin D, protects against multiple cancers through unelucidated mechanisms. The oncogenic long non-coding RNA (lncRNA) CCAT2 (colon cancer associated transcript 2) is overexpressed in ovarian cancer. Here, we foundd that calcitriol inhibited CCAT2 expression in ovarian cancer cell lines. Treatment with calcitriol inhibited ovarian cancer cell proliferation, migration, and invasion. As a result of CCAT2 inhibition, calcitriol decreased the binding of transcription factor TCF7L2 (TCF4) to the *MYC* promoter, resulting in the repression of c-Myc protein expression. Our results suggest a novel anti-cancer mechanism of vitamin D by targeting CCAT2 in ovarian cancer. The findings may help develop vitamin D as a practical and inexpensive nutraceutical for ovarian cancer prevention.

## 1. Introduction

Ovarian cancer is one of the most lethal gynecologic cancers causing around 13,940 deaths in 2020 (American Cancer Society), which accounts for 5% cancer deaths among women [1,2]. Over 70% of ovarian cancer patients are diagnosed in advanced stages (III and IV) with less than 30% five-year survival rates [1,3,4]. Such poor clinical outcomes are mainly caused by late diagnosis and cancer resistance to chemotherapy, which leads to disease progression with highly aggressive metastasis [5]. Therefore, to achieve better clinical outcomes, there is an imperative need to identify new biomarkers for early diagnosis and new prevention strategies.

Extensive studies have reported that long non-coding RNAs (lncRNAs), the non-coding RNA transcripts larger than 200 nucleotides, can be used as diagnostic biomarkers and therapeutic targets due to their involvement in many hallmarks of cancers [6,7]. CCAT2 (Colon cancer-associated transcript 2), a novel lncRNA initially identified by Ling et al. in colorectal cancer, has been shown to promote tumor growth and liver metastasis [8]. The subsequent studies have reported that CCAT2 is positively associated with distant metastasis of non-small cell lung cancer [9], breast cancer [10,11], ovarian cancer [1,12,13], cervical cancer [14,15] and gastric cancer [16,17], which suggests that CCAT2 could be a prognostic biomarker for tumor metastasis. Overexpression of CCAT2 promotes ovarian tumor progression by acting as a miRNA sponge [12] or stimulating the Wnt/β-catenin signaling pathway [13]. CCAT2 is located at 335kb centromeric upstream of the *MYC* oncogene, exerting strong effect on *MYC* gene expression via *cis* and *trans* activities [8,18]. Thus, in addition to being a prognostic biomarker, CCAT2 can also serve as a potential therapeutic target.

Epidemiological studies have indicated that the incidence and mortality rates of ovarian cancer are higher in area with lower sunlight exposure [19,20]. Solar ultraviolet-B (UVB) irradiation increases the serum vitamin D level in humans [21,22], suggesting that vitamin D may have protective effect against ovarian cancer. Calcitriol (1,25-dihydroxycholecaldiferol, 1,25(OH)_2_D_3_), the active form of vitamin D, exerts antineoplastic effects in colorectal cancer [23], prostate cancer [24,25], ovarian cancer [26,27] and breast cancer [28]. A Phase II trial of the combinational therapy with calcitriol and anti-inflammatory drugs [29,30] (such as naproxen and dexamethasone) showed antitumor activities in prostate cancer patients with safety and well tolerance. Moreover, Phase I studies also found that calcitriol could potentiate the cytotoxicity of paclitaxel [31] in patients with advanced cancer. However, the underlying anti-cancer mechanisms of calcitriol remain unclear. Calcitriol interacts with vitamin D receptor (VDR) protein and then partners with retinoid X receptor (RXR). The calcitriol-VDR-RXR complex binds to vitamin D response element (VDRE) at specific regions of genomic DNA [32]. By regulating the expression of vitamin D responsive genes, calcitriol participates in several important signaling pathways in cancer cells [33]. Emerging studies reveal that many VDREs have been found in non-coding regions [34,35,36], which suggests that vitamin D may modulate the transcriptional/post-transcriptional process of cancer-related genes. Studies on the non-coding regions likely to reveal the hidden mechanism of vitamin D on cancer prevention [36,37].

In this study, we found that calcitriol suppressed ovarian cancer cell growth, migration, and invasion by down-regulating lncRNA CCAT2, thus inhibiting its downstream onco-protein c-Myc. Our results provide a novel target, CCAT2, in the mechanism of action of vitamin D. The data strengthen the support of vitamin D as a key nutraceutical to prevent ovarian cancer invasion and metastasis.

## 2. Results

### 2.1. CCAT2 and MYC Genes are Overexpressed in Ovarian Cancer

**Figure 1:**
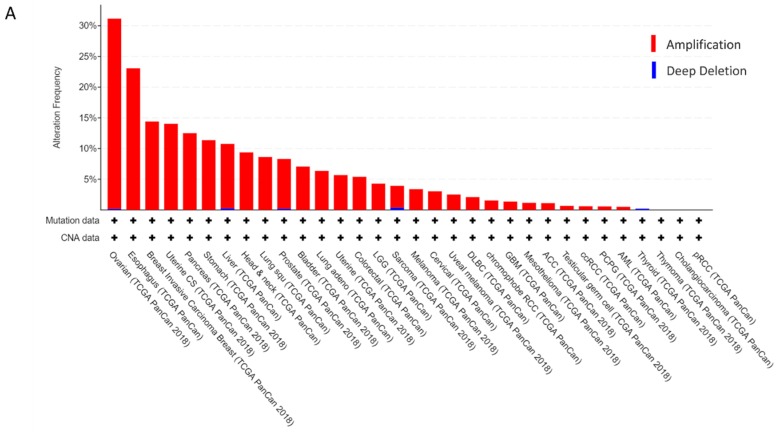

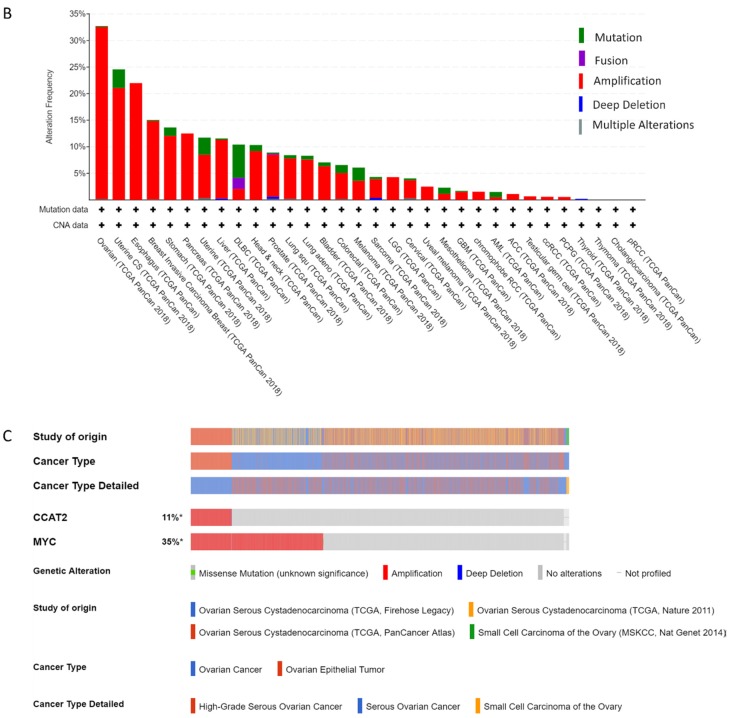
CCAT2 and *MYC* are most frequently altered in ovarian cancer samples against cross-cancer studies. (**A**) CCAT2 amplification frequencies are the highest in ovarian cancer samples. (**B**) The frequencies of *MYC* gene amplification are the highest in ovarian cancer samples. (**C**) The genomic alterations in a query of two genes CCAT2 and *MYC* across a set of ovarian cancer samples. Each row represents a gene. Each column represents an ovarian cancer sample. Red bars stand for gene amplifications, blue bars indicate deep deletions, and green squares are missense mutations.

Based on analysis of the data retrieved from an online database cBioPortal (https://www.cbioportal.org/), we found that CCAT2 and *MYC* genes are most frequently amplified in ovarian epithelial cancers (Figure 1A,B). Among 1680 patients with ovarian cancers, about 185 cases (11%) have the alterations in CCAT2 and 588 cases (35%) have the alterations in *MYC* (Figure 1C), indicating a high incidence of the aberrant expression of CCAT2 and *MYC* in ovarian epithelial carcinomas. For CCAT2, most of the alterations are amplifications and a few deep deletions. For *MYC*, most of the alterations are amplifications and a few mutations, fusions and deep deletions [38,39].

### 2.2. Calcitriol Down-Regulated CCAT2 in Ovarian Cancer Cell Lines

**Figure 2:**
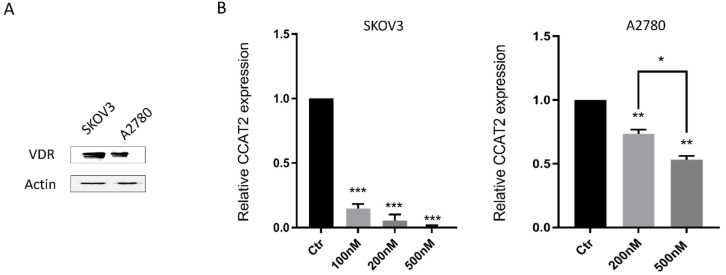
Calcitriol down-regulated CCAT2 in ovarian cancer cells. (**A**) VDR protein is detected in SKOV3 and A2780 cell lines by western blotting. (**B**) The CCAT2 expression level are significantly down-regulated by calcitriol in a dose-dependent manner. All experiments have been repeated three times and data shown are mean values ± SD, (*) *p* < 0.05; (**) *p* < 0.01; (***) *p* < 0.001.

As a critical mediator of the anti-cancer activity of vitamin D, VDR is highly expressed in ovarian tumor tissues [40]. To determine the effects of vitamin D in ovarian cancer cells, we first examined the expression level of VDR protein in ovarian cancer cell lines, SKOV3 and A2780. We found that VDR is expressed in both SKOV3 and A2780 cells (Figure 2A). Since CCAT2 expression is elevated in ovarian cancer (Figure 1A) and CCAT2 promotes cancer growth and metastasis, we determined the effects of vitamin D on CCAT2 in these cell lines. Interestingly, CCAT2 was down-regulated by calcitriol in both SKOV3 and A2780 cells in a dose-dependent manner (Figure 2B).

### 2.3. Calcitriol Inhibited the Proliferation and Changed Cell Cycle Distribution of Ovarian Cancer Cells

**Figure 3:**
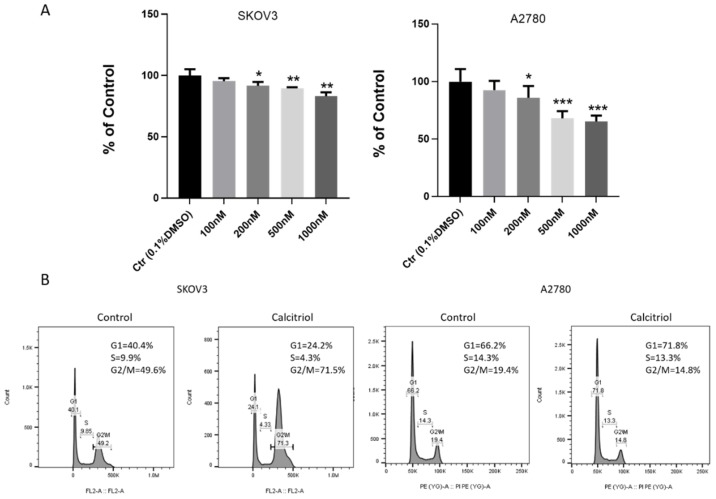
Calcitriol inhibited the *in vitro* growth and changed cell cycle distribution of ovarian cancer cells. (**A**) Calcitriol inhibited the cell growth of SKOV3 and A2780 cells. Cell proliferation was examined after 3-day treatment using WST-1 assay as described in the Materials and Methods section. The inhibition percentage was normalized by the control group. (**B**) Calcitriol increased the percentage of G2/M phase in SKOV3 cells and increased the percentage of G1 phase in A2780 cells. The cell cycle distribution was analyzed using flow cytometry. All experiments have been repeated three times and data shown are mean values ± SD, (*) *p* < 0.05; (**) *p* < 0.01; (***) *p* < 0.001.

We examined the effect of calcitriol on ovarian cancer cell proliferation. Using WST-1 assay, we found that compared to vehicle (0.1% DMSO), calcitriol attenuated the cell growth of both SKOV3 and A2780 cells in a dose-dependent manner (Figure 3A). At a concentration of 200 nM or higher, the inhibitory effect on ovarian cancer cell growth was of statistical significance. We also analyzed the cell cycle distribution of vehicle and calcitriol-treated cells by flow cytometry (Figure 3B). We found that calcitriol significantly increased the percentage of SKOV3 cells in G2/M phase, and it slightly increased the percentage of A2780 cells in G1 phase. The SKOV3 cells in G2/M phase increased from 49.6% to 71.5% after calcitriol treatment. For A2780 cells, the percentage in G1 phase increased from 66.2% to 71.8% in the calcitriol-treated group.

### 2.4. Calcitriol Suppressed the Migration and Invasion of Ovarian Cancer Cells

**Figure 4:**
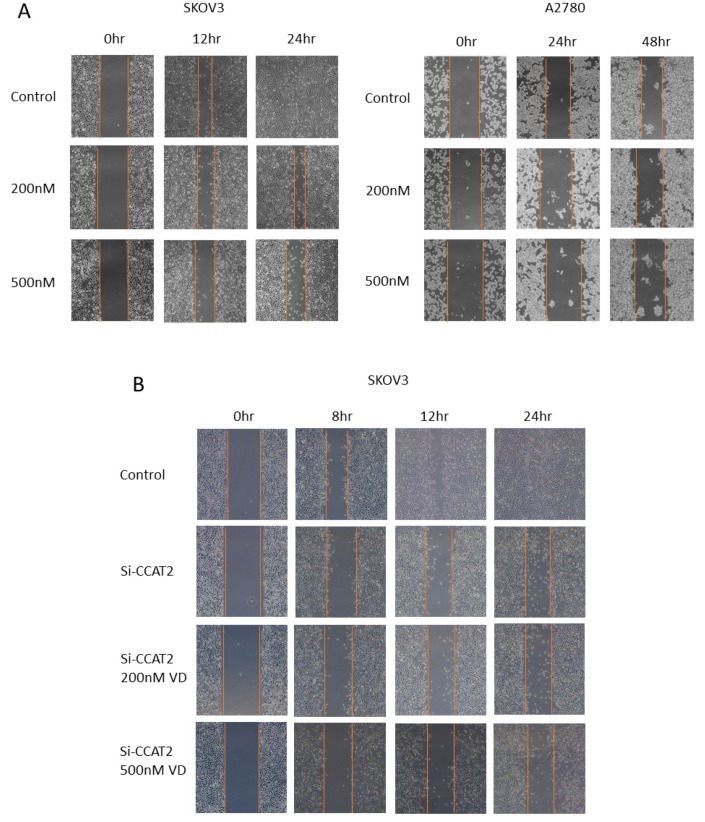
Calcitriol inhibited the directional migration of ovarian cancer cells. (**A**) Calcitriol inhibited the movement to the scratched area by SKOV3 and A2780 cells in a dose-dependent manner. (**B**) SKOV3 cells were transfected with siRNA targeting CCAT2. Following siRNA transfection, cells were treated with 200 nM and 500 nM calcitriol, respectively. The wound closure was observed using a microscope (scale bars: 200 µm).

**Figure 5:**
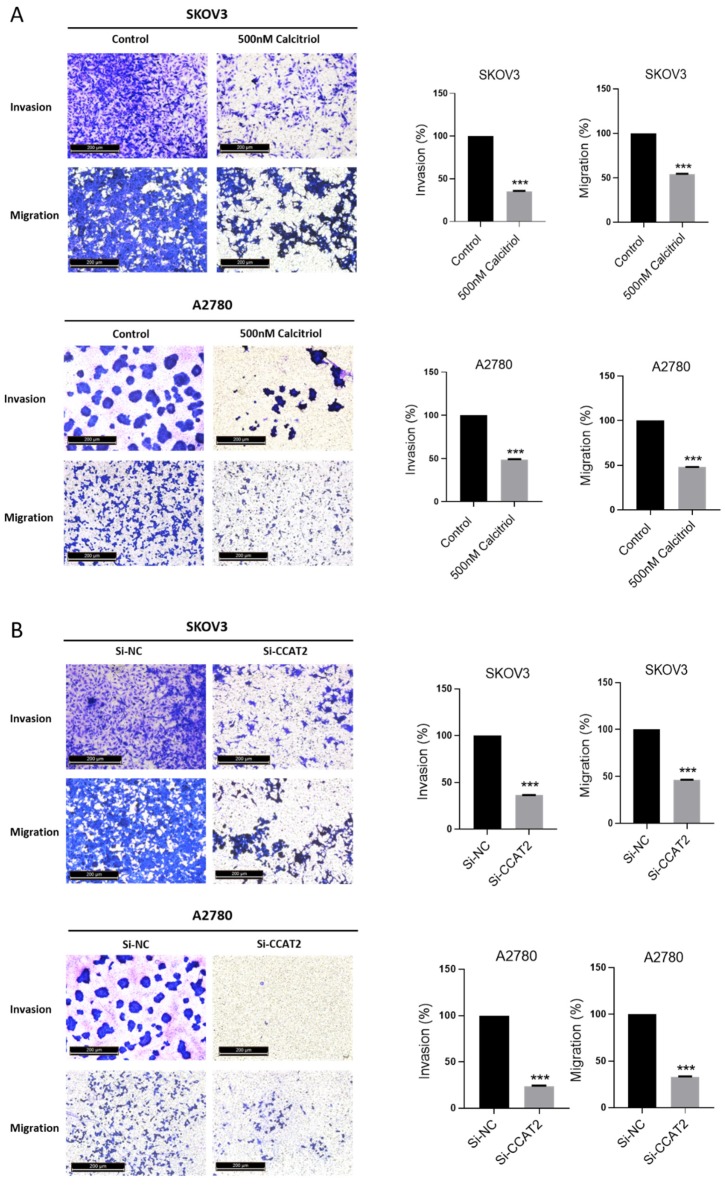
Calcitriol suppressed the migration and invasion of ovarian cancer cells. (**A**) SKOV3 and A2780 cells were treated with vehicle (0.1% DMSO) and 500nM calcitriol. (**B**) SKOV3 and A2780 cells were transfected with siRNA targeting CCAT2. The migration and invasion capacities were assessed using transwell assay. After resolving crystal violet into 33% glacial acetic acid, the invaded cell numbers of each well were quantified by absorbance values at 590 nm. (***) *p* < 0.001.

To assess the effects of calcitriol on migration and invasive capacity of ovarian cancer cells, we conducted the wound healing and transwell assays. The results of wound healing showed that calcitriol inhibited the directional migration of ovarian cancer cells in a dose-dependent manner. Calcitriol slowed the directional closure of both SKOV3 and A2780 cells in a dose-dependent manner (Figure 4A). In SKOV3 cells, the wound distance of control group became very narrow after 12 h and the wound almost disappeared at 24 h, reflecting the high migration ability of SKOV3 cells. In contrast, there was still a wide wound distance in the calcitriol-treated cells at 24 h. To determine the role of CCAT2 in cell migration, we used siRNA to specifically knockdown the levels of CCAT2. As shown in Figure 4B, after siRNA knockdown of CCAT2, the wound closure of SKOV3 cells was significantly inhibited at 12 h. Intriguingly, after the siRNA knockdown of CCAT2, additional treatment with calcitriol no longer further inhibit the migration of SKOV3 cells, whether or not the dose of calcitriol was increased. The data suggest that calcitriol inhibits the migration of ovarian cancer cells mainly through suppressing the function of CCAT2. We further determined the migration and invasion abilities of the cancer cells by transwell assays. The results showed that calcitriol significantly inhibited the migration and invasion of both SKOV3 and A2780 cells (Figure 5A). In addition, siRNA knockdown of CCAT2 significantly inhibited the migration and invasion in both cell lines (Figure 5B). On the other hand, overexpression of CCAT2 increased the invasion of A2780 cells (Figure S1). Comparing to the effects of calcitriol on cell proliferation, 500nM calcitriol only inhibited 10% of SKOV3 cell proliferation after 3-day treatment (Figure 3A), while we observed the effects of 500nM calcitriol on the directional migration at 24 h (Figure 4A), suggesting the difference in migration is not a result of growth inhibition by calcitriol.

### 2.5. Calcitriol Inhibits c-Myc Expression by Targeting CCAT2 in Ovarian Cancer Cells

**Figure 6:**
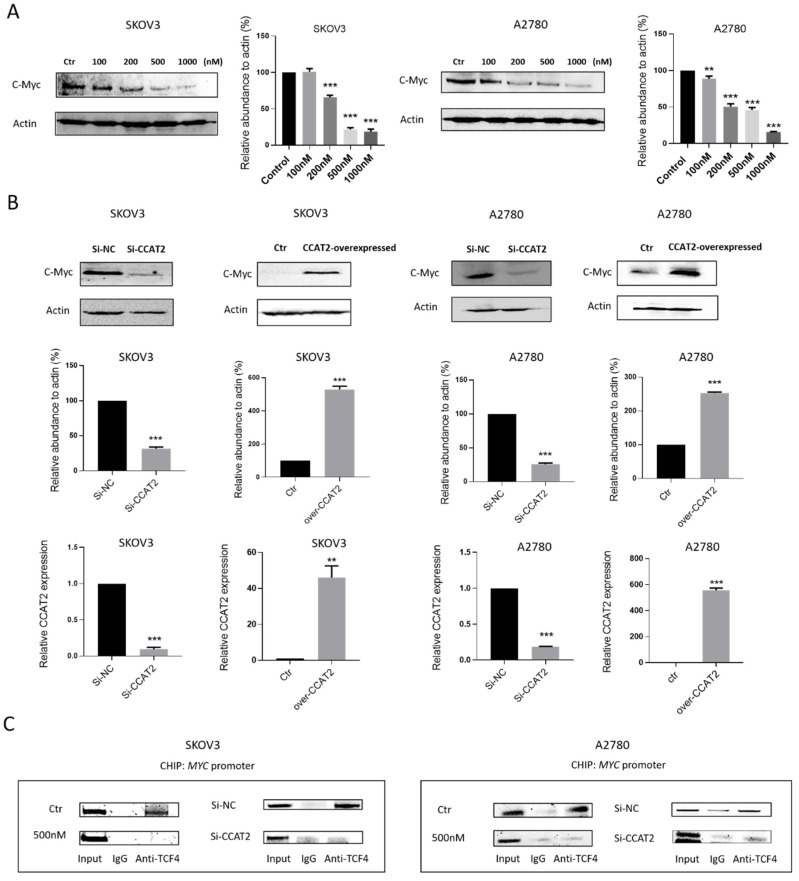
Calcitriol inhibited the expression of c-Myc by down-regulation of CCAT2. (**A**) SKOV3 and A2780 cells were treated with vehicle (0.1% DMSO), 100 nM, 200 nM, 500 nM and 1000 nM of calcitriol for 48 h. The expression level of c-Myc protein was examined using Western blot. (**B**) SKOV3 and A2780 were transfected with CCAT2 targeting siRNA or CCAT2-overexpressed plasmid. After 24 h, the level of CCAT2 was determined using Real-Time PCR. After 48 h, the expression level of c-Myc protein was examined using Western blot. (**C**) SKOV3 and A2780 cells were transfected with CCAT2 targeting siRNA or treated with 500 nM calcitriol. Binding of TCF4 to the *MYC* promoter was detected using ChIP assays as described in the Materials and Methods section. The expression level of *MYC* promoter was quantified using electrophoretic gels. (**) *p* < 0.01; (***) *p* < 0.001.

As previously reported, lncRNA CCAT2 promotes cancer progression by increasing *MYC* gene expression in cancer cells [8]. Given the negatively regulatory effect of calcitriol on CCAT2, we studied whether calcitriol also inhibits the expression of c-Myc protein in ovarian cancer cells. We found that c-Myc protein expression was inhibited by calcitriol in a dose-dependent manner in both SKOV3 and A2780 cells (Figure 6A). To determine the role of CCAT2 in calcitriol’s inhibition of c-Myc protein, we used siRNA to specifically knock down CCAT2 in ovarian cancer cells. In both SKOV3 and A2780 cells, siRNA knockdown of CCAT2 caused a decrease in CCAT2 and c-Myc expression. In contrast, when CCAT2 was overexpressed exogenously, the expression level of CCAT2 was significantly increased, and consequently, c-Myc protein level was increased in SKOV3 and A2780 cells (Figure 6B). CCAT2 regulates *MYC* expression by interacting with transcription factor TCF7L2 (TCF4) to activate the promoter of *MYC* gene [8,18]. To explore the underlying mechanism of calcitriol regulation of *MYC* in ovarian cancer cells, we studied the interaction of CCAT2 and TCF4 at the *MYC* promoter. Using chromatin immunoprecipitation (ChIP) assay, we found that in both SKOV3 and A2780 cells, siRNA knockdown of CCAT2 or calcitriol treatment caused a decrease in the binding of TCF4 to the *MYC* promoter (Figure 6C). The results suggest that calcitriol inhibits c-Myc expression partly through down-regulating CCAT2 and interfering the binding of TCF4 to the *MYC* promoter.

## 3. Discussion

Ovarian cancer is a deadly disease and its low five-year survival rate is due to the aggressive nature of metastasis. Once the cancer metastasizes, it is resistant to chemotherapy drugs and there is no effective treatment. In this study, we found that a low dose of calcitriol has a modest inhibitory effect on ovarian cancer cell proliferation. Notably, calcitriol exerts significant activity against the migration and invasion of ovarian cancer cells. The results suggest that vitamin D treatment may be a practical and inexpensive method to prevent ovarian tumor metastasis. A long-term animal experiment is needed to study whether calcitriol can prevent *in vivo* tumor growth and distant metastasis. Our *in vitro* study showed that as low as 100 nM calcitriol can significantly down-regulate CCAT2 and 500 nM calcitriol has obvious anti-growth and anti-invasion effects in ovarian cancer cell lines. As one pharmacokinetic study reported, the Cmax of calcitriol in plasma could reach 100 nM at the dose of 0.5 μg/mouse intraperitoneally [41]. Another study suggests that after intravenous administration of 50 μg/kg calcitriol, drug concentration could reach over 500 nM within 2 h [42]. Based on these PK studies, it will be useful to perform *in vivo* efficacy study to determine the effects of calcitriol against tumor growth and metastasis in subcutaneous or orthotopic xenograft mouse models. To this end, an orthotopic ovarian cancer mouse model would be the most suitable to evaluate the anti-metastasis activity of calcitriol [43,44]. In addition, to reach a higher drug concentration in the tumor site, drug delivery systems, such as nanoparticles or micelle, could be applied to improve the solubility of calcitriol and delivery to the tumors [45,46].

Our results show that calcitriol decreases lncRNA CCAT2 expression in ovarian cancer cells. To our knowledge, this is the first report on vitamin D-regulated lncRNA in ovarian cancer. As CCAT2 has been shown to promote cancer growth and metastasis in several types of cancers, our findings that vitamin D inhibits CCAT2 shed light on a novel mechanism of how vitamin D can prevent cancer metastasis. By disrupting the interaction between CCAT2 and transcription factor TCF4 (a key transcription factor in WNT signaling), vitamin D decreases the highly oncogenic c-Myc protein by inactivating the *MYC* promoter. It was reported before that vitamin D inhibits *MYC* gene in cancer cells [47], although the mechanism was not clear. Our data offer a new insight on the mechanism of vitamin D-induced decrease in c-Myc in ovarian cancer.

The mechanism of how calcitriol decreases CCAT2 expression is not clear. One possible mechanism is that the CCAT2 promoter contains the VDRE sequence and calcitriol inhibits CCAT2 expression by directly acting on the CCAT2 promoter. Alternatively, calcitriol may inhibit CCAT2 expression by regulating the transcription factors that control CCAT2 expression. It has been reported that transcription factor E2F1 binds to and activates the CCAT2 promoter [48]. It is possible that vitamin D may down-regulate CCAT2 by inhibiting E2F1—a reported target of vitamin D [49]. The detail mechanism of CCAT2 suppression by vitamin D can be illustrated in future studies.

## 4. Materials and Methods

### 4.1. Cell Culture

The epithelial ovarian carcinoma (EOC) cell lines SKOV3 and A2780 were kindly provided by Dr. Jinsong Liu’s lab at M.D. Anderson Cancer Center (Houston, TX, USA). The SKOV3 cells have been verified by short-tandem repeat (STR) profiling in Dr. Jinsong Liu’s lab. Cells were cultured in RPMI-1640 medium (Corning, NY, USA) supplemented with 10% fetal bovine serum (Atlantic, Minneapolis, MN, USA) and 1% penicillin-streptomycin (Corning, NY, USA).

### 4.2. Cell Proliferation Assay

Cell proliferation assay was performed using WST-1 cell proliferation assay kit (Takara, Mountain View, CA, USA) according to the instruction. 4 × 10^3^ SKOV3 and A2780 cells were seeded into each well of 96-well cell culture plates with six replicates wells for each group. The cells were treated by vehicle (0.1% DMSO), 100 nM, 200 nM, 500 nM and 1000 nM calcitriol for 3 days, respectively. At the end of treatment, 1:10 Premix WST-1 was added into each well and incubated for 2 h at 37 °C in the dark. The optical absorbance values were measured at 450 nm using Cytation 5 reader (BioTek, Winooski, VT, USA).

### 4.3. Cell Cycle Assay

Cell cycle distribution was conducted using propidium iodide flow cytometry kit (Abcam, Cambridge, MA, USA). First, 10^6^ SKOV3 and A2780 cells were seeded into each well of 6-well culture plates with triplicate wells for each group. The cells were treated by vehicle (0.1% DMSO) and 200 nM calcitriol for 3 days. At the end of treatment, the cells were harvested, washed with PBS, and fixed in 70% cold ethanol at −20 °C for 1 h. Then, the fixed cells were washed with PBS and applied in 500 μL 1× filtered PBS + 1× propidium iodide (PI) + 20× RNase staining solution at 37 °C in the dark for 30 min. The PI-stained cells were analyzed by BD FACS Melody cell sorter and BD Accuri C6 Cytometer (BD Biosciences, San Jose, CA, USA).

### 4.4. Small Interfering RNAs (siRNAs) Transfection

10^4^ SKOV3 or A2780 cells were transfected with ~40 pmoL siRNAs targeting CCAT2 (Cat#: R-191047-00-0005; Dharmacon) or negative control siRNA (sc-37007, Santa Cruz, Dallas, TX, USA), together with X-treme reagent (Thermo Fisher Scientific, Waltham, MA, USA) and Opti-MEM reduced serum medium according to the manufacturer’s protocol.

### 4.5. Monolayer Wound Healing Assay

The directional migration ability of ovarian cancer cells was studied using wound healing assay. First, 10^6^ SKOV3 and A2780 cells were seeded into each well of 6-well culture plates. When cells reached 80% density, wounds were created by scratching the surface of each cells with a 100 μL pipette tip. Then, cells were treated by vehicle (0.1% DMSO), 200 and 500 nM calcitriol, respectively. Images of the original wounds and the movement of the cells into the scratched area were captured at 0 h, 12 h, 24 h and 48 h using a microscope (5× objective; Leica, Buffalo Grove, IL, USA). For siRNA transfection, 2 × 10^5^ SKOV3 cells were seeded into each well of 6-well culture plates. Cells were transfected with siRNA targeting CCAT2 or negative control (Si-NC), respectively. When cells reached 80% density, wounds were created by scratching the surface of each cells with a 100 μL pipette tip. Images of the original wounds and the movement of the cells into the scratched area were captured at 0 h, 8 h, 12 h and 24 h using microscope (5× objective; Leica, USA). All experiments were repeated three times.

### 4.6. Transwell Invasion and Migration Assay

The invasion assay was conducted using transwell chambers (8-µm pore size; Costar, Corning, USA). Matrigel was coated onto the upper side of the upper chambers at 37 °C for at least 1 h. Then, 10^5^ SKOV3 and A2780 cells were seeded in the upper chambers with 200 μL FBS-free medium. The lower chambers were filled with 750 μL 10% FBS medium. Cells were treated by vehicle (0.1% DMSO) and 500nM calcitriol. After incubation for 48 h, the invaded cells were fixed on the bottom surface of the upper chambers using 4% formaldehyde and 100% methanol and then stained with 0.05% crystal violet for 5 min at room temperature. The images of five random fields were captured using microscope (10× objective; Leica; USA). The invaded cell numbers were quantified by absorbance values at 590 nm after resolving crystal violet into 33% glacial acetic acid (EMD, Millipore Corporation, Burlington, MA, USA). The migration assay was also conducted using transwell chambers (8-µm pore size; Costar, Corning, NY, USA). First, 10^5^ SKOV3 and A2780 cells were seeded in the upper chambers with 200ul FBS-free medium. The lower chambers were filled with 750 μL 10% FBS medium. Cells were treated by vehicle (0.1% DMSO) and 500 nM calcitriol. After incubation for at least 24 h, the migrated cells were fixed on the bottom surface of the upper chambers and stained with 0.05% crystal violet. The images of five random fields were captured using microscope (10× objective; Leica; USA). The migrated cell numbers were quantified by absorbance values at 590 nm after resolving crystal violet into 33% glacial acetic acid (EMD, Millipore Corporation, Burlington, MA, USA).

### 4.7. Total RNA Isolation, cDNA Synthesis, and Quantification

1.5 × 10^5^ SKOV3 and A2780 cells were seeded into each well of 6-well culture plates. Cells were treated by vehicle (0.1% DMSO), 200 nM and 500 nM calcitriol for 48 h, respectively. Cells were harvested and total RNA was extracted using RNeasy mini kit (QIAGEN, Hilden, Germany). RNA was reversely transcribed into cDNA using M-MLV reverse transcription system (Promega, Madison, WI, USA) and performed to Real-Time PCR using Taqman gene expression assay CCAT2 (assay ID: Hs04403001_s1, Thermo Fisher Scientific, USA) and 18S (assay ID: Hs99999901_s1; Thermo Fisher Scientific, Waltham, MA, USA), together with Taqman master Universal mix II (Thermo Scientific). The relative abundance of RNA was calculated using 2^−∆∆*C*T^ method compared with 18S expression.

### 4.8. Western Blotting

For the Western blotting, 1.5 × 10^5^ SKOV3 and A2780 cells were seeded into each well of 6-well culture plates. Cells were treated by vehicle (0.1% DMSO), 200 nM and 500 nM calcitriol for 48 h, respectively. Cells were harvested and lysed in RIPA + protein inhibitor lysis buffer. The supernatant containing protein were collected and protein concentration was determined by Bio-spectrometer (Eppendorf). Protein samples were separated by SDS-PAGE and electro-transferred onto nitrocellulose blotting membrane (GE healthcare). After blocking, membranes were incubated overnight at 4 °C with 1:500 c-Myc antibody (Santa Cruz) and actin (Invitrogen) separately. The goat anti-mouse secondary antibody was used to blot target proteins. Signals were detected using chemiluminescence ECL detection (advansta) and ChemiDoc imaging system (Bio-RAD).

### 4.9. The Chromatin Immunoprecipitation (ChIP) Assay

The ChIP assay was carried out with Chromatin immunoprecipitation assay kit (EMD, Millipore Corporation, USA) according to manufacturer’s protocol. First, 10^6^ SKOV3 and A2780 cells were cross-linked with 4% formaldehyde at 37 °C for 10 min and ended the crosslink with glycine for 5 min. The cells were collected using cell scraper and the genomic DNA was broken into small pieces using ultra-Sonicator (Fisher Scientific, Winooski, VT, USA). An amount of 100 μL was kept as input reference. Immunoprecipitation was incubated with anti-TCF4 antibody (Santa Cruz, Dallas, TX, USA) or normal mouse IgG overnight at 4 °C with gentle rotating and then applied with protein G Agarose beads (EMD, Millipore Corporation, USA) for at least 1 h. The collected beads were washed using salt buffer and TE buffer. Then, the DNA-protein complex was eluted from the beads using elution buffer. The DNA-protein crosslink was reversed by 5 M NaCI, EDTA, Tris-HCL (pH 6.5) and Proteinase K at 65 °C for 2 h. The DNA was collected and purified using QIAquick PCR purification kit (QIAGEN, USA). The immunoprecipitated DNA was amplified by PCR using specific primers: *MYC* promoter (F), 5′-TTCTCCCAAACCCGGCAGCC-3′; *MYC* promoter (R), 5′-GAGGCGTCTGTTTAGCCCTG-3′. In each PCR reaction, the corresponding inputs were taken in parallel for controls. The PCR products were subjected to 2.5% agarose gel electrophoresis.

### 4.10. Statistical Analysis

All statistical analyses were performed using GraphPad Prism 7. All the data were presented as mean value ± SD. The statistically significant differences among different groups were assessed using one-way ANOVA complemented with Student’s *t*-test. *p*-value < 0.05 were considered to indicate statistically significance.

## 5. Conclusions

Our study identified a novel lncRNA-associated mechanism for the anti-cancer activity of vitamin D in ovarian cancer cells. As shown in the model, calcitriol suppresses ovarian cancer cell growth, migration and invasion by down-regulating CCAT2 and repressing its interaction with TCF4, decreasing CCAT2/TCF4 binding to the *MYC* promoter. Our findings suggest CCAT2 as a potential target for metastasis prevention in ovarian cancer.

## Supplementary Materials

Supplementary materials can be found at https://www.mdpi.com/1422-0067/21/7/2334/s1.
