# Supplementary material for: Vitamin D Suppresses Ovarian Cancer Growth and Invasion by Targeting Long Non-Coding RNA CCAT2
Source: Int J Mol Sci. 2020 Mar 27;21(7):2334. doi: 10.3390/ijms21072334 (PMC7177268; doi:10.3390/ijms21072334)
Supplement: Supplementary file 1 [file ijms-21-02334-s001.pdf]

Supplementary Materials

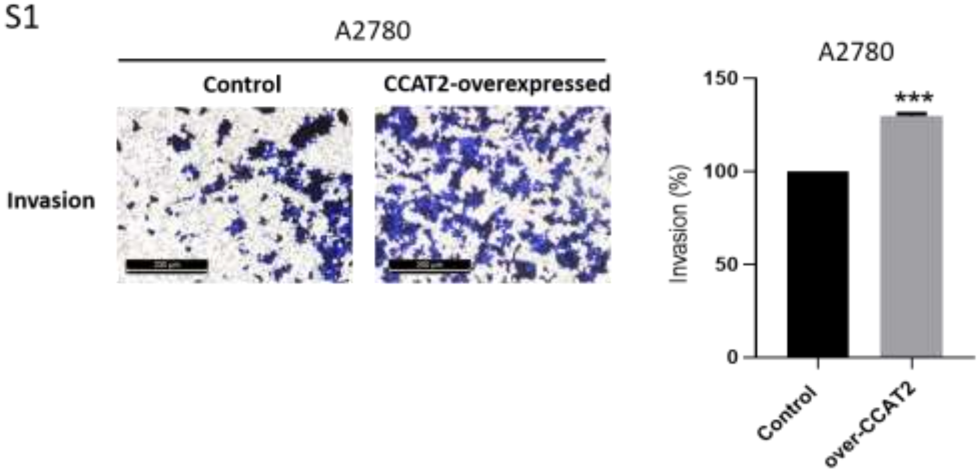

Figure S1. CCAT2 promotes cell invasion in A2780 cells. A2780 cells were transfected with PCS6-CCAT2 plasmid and performed to the invasion assay for 48 hours. The invasive capability of CCAT2-overexpressed A2780 cells was obviously induced compared to the control group. (***) $p < 0.001$
